# ASSOCIATION BETWEEN HOME HEALTH SERVICE USE AND FACILITY ADMISSION IN OLDER ADULTS WITH AND WITHOUT DEMENTIA

**DOI:** 10.1093/geroni/igz038.3224

**Published:** 2019-11-08

**Authors:** Jinjiao Wang, Thomas V Caprio, Adam Simning, jingjing Shang, Yeates Conwell, Fang Yu, Yue Li

**Affiliations:** 1 University of Rochester, Rochester, New York, United States; 2 . University of Rochester Medical Center, Department of Psychiatry, NY, Rochester, New York, United States; 3 Columbia University School of Nursing, New York, New York, United States; 4 University of Rochester School of Medicine, Rochester, New York, United States; 5 University of Minnesota School of Nursing, Minneapolis, Minnesota, United States; 6 University of Rochester, Department of Public Health Sciences, NY, Rochester, New York, United States

## Abstract

This study is a secondary analysis of the Outcome and Assessment Information Set (OASIS) and administrative billing records of 6,153 adults ≥ 65 years old who received home health (HH) from a not-for-profit HH agency in upstate New York in CY 2017. We evaluated the association between the use of home health services (HH) with the hazard of unplanned facility admissions among Medicare patients with and without Alzheimer’s disease and related dementia (ADRD). Outcome was time from HH start of care to an unplanned facility admission of any type, including the hospital, nursing home, or rehabilitation facility. Independent variables included weekly intensity (visits/week, hours/week) of each discipline, including skilled nursing (SN), physical therapy (PT), occupational therapy (OT), social work (SW), and home health aide (HA), separately. ADRD was identified by diagnosis (ICD-10 codes) and cognitive impairment (M1700, M1710, M1740 [OASIS]). In multivariable Cox Proportional hazard models that adjusted for time-varying effects of HH intensity, receiving the highest intensity of SN (3.3 visits of 2.78 hours per week) and PT (2.5 visits of 2 hours per week) was related to up to a 54% and 86% decrease, respectively, in the hazard of unplanned facility admission among patients with ADRD (n=1,525), and decreases of 56% and 90% respectively among patients without ADRD (n=4,628). This is the first study in the United States showing that receiving a sufficient amount and appropriate mix of HH services was associated with substantially reduced risk of unplanned facility admission among patients with ADRD by up to 86%.

